# P-200. Preliminary Results from a Multistate *C. difficile* Surveillance Network: The Surveillance Highlighting Existing/Emerging Resistance Limiting Our *C. difficile* Knowledge (SHERLOCK) Project

**DOI:** 10.1093/ofid/ofae631.404

**Published:** 2025-01-29

**Authors:** Travis J Carlson, Reeham Saleh, Jonah Dixon, Colleen N Riley, Taryn A Eubank, Anne J Gonzales-Luna, M Jahangir Alam, Khurshida Begum, Nasia Safdar, Kevin W Garey

**Affiliations:** The University of Texas at Austin College of Pharmacy, San Antonio, Texas; University of Houston College of Pharmacy, Houston, Texas; University of Wisconsin - Madison, Madison, Wisconsin; University of Wisconsin - Madison, Madison, Wisconsin; University of Houston College of Pharmacy, Houston, Texas; Department of Pharmacy Practice and Translational Research, University of Houston College of Pharmacy, Houston, TX; Department of Pharmacy Practice and Translational Research, University of Houston College of Pharmacy, Houston, Texas, USA, Houston, Texas; Department of Pharmacy Practice and Translational Research, University of Houston College of Pharmacy, Houston, Texas, USA, Houston, Texas; University of Wisconsin School of Medicine and Public Health, Madison, WI; University of Houston, Houston, TX

## Abstract

**Background:**

*C. difficile* infection is unique in that the diagnosis is made via the identification of toxins in stool rather than by bacterial growth. Therefore, strain typing and antibiotic susceptibility testing are not routinely performed on *C. difficile* isolates. Instead, active surveillance programs are required to identify new epidemic and/or antibiotic-resistant strains. The purpose of this project was to create a comprehensive, multistate surveillance program that can quantify the variability in *C. difficile* strain prevalence and antibiotic resistance across the United States.

**Table 1.**
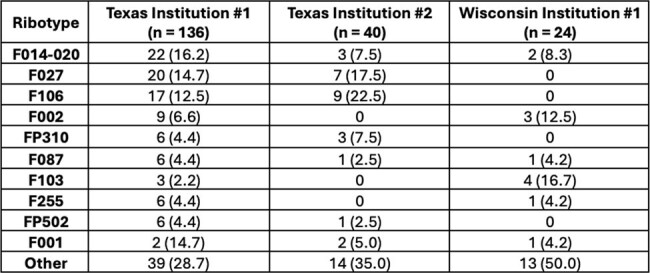
C. difficile Ribotypes from Three Institutions in Two States

Results are reported as number (proportion)

**Methods:**

All institutions participating in The Surveillance Highlighting Existing/Emerging Resistance Limiting Our *C. difficile* Knowledge (SHERLOCK) Project were asked to contribute a minimum of 40 leftover, *C. difficile*-positive stool samples each calendar year. The collection of stools for *C. difficile* testing was conducted as part of routine clinical care. All isolates were sent to the University of Houston for fluorescent polymerase chain reaction (PCR) ribotyping and antibiotic susceptibility testing.

**Results:**

Between January 2021 and December 2023, institutions from three states (Iowa, North Carolina, and Wisconsin) contributed a total of 345 *C. difficile*-positive stool samples. Of those, 24 isolates from one institution have undergone fluorescent PCR ribotyping and were compared to 176 isolates previously collected in Texas between January 2018 and December 2021 (Table 1). The three most prevalent ribotypes in Texas were F027 (15.3%), F106 (14.8%), and F014-020 (14.2%). Conversely, the three most prevalent ribotypes in Wisconsin were F103 (16.7%), F002 (12.5%), and F014-020 (8.3%). Notably, two known, virulent ribotypes, RT027 and RT106, were absent from Wisconsin isolates.

**Conclusion:**

These preliminary data demonstrate differences in *C. difficile* strain prevalence by state, which highlights the importance of a comprehensive, multistate surveillance program.

**Disclosures:**

**Travis J. Carlson, PharmD, BCIDP**, Aimmune Therapeutics, Inc.: Speakers Bureau **Anne J. Gonzales-Luna, PharmD, BCIDP**, Ferring Pharmaceuticals: Advisor/Consultant|Innoviva Specialty Therapeutics: Advisor/Consultant|Merck and Co: Grant/Research Support|Paratek Pharmaceuticals: Grant/Research Support|Seres Therapeutics: Grant/Research Support **Kevin W. Garey, PharmD, MS**, Acurx: Grant/Research Support

